# Transcriptomic Insights into the Developmental Dynamics of *Eimeria acervulina*: A Comparative Study of a Precocious Line and the Wild Type

**DOI:** 10.3390/genes15070831

**Published:** 2024-06-24

**Authors:** Ning Zhang, Xiaojin Li, Jie Liu, Linlin Chen, Sixin Zhang, Xianyong Liu, Xinming Tang, Xun Suo, Yuanyuan Zhang

**Affiliations:** 1 National Key Laboratory of Veterinary Public Health Security, Key Laboratory of Animal Epidemiology and Zoonosis of Ministry of Agriculture, National Animal Protozoa Laboratory, College of Veterinary Medicine, China Agricultural University, Beijing 100193, China; 13285920591@163.com (N.Z.);; 2 Institute of Animal Science, Chinese Academy of Agricultural Sciences, Beijing 100193, China; 3 Key Laboratory of Animal Genetics, Breeding and Reproduction of the Ministry of Agriculture and Beijing Key Laboratory of Animal Genetic Improvement, China Agricultural University, Beijing 100193, China

**Keywords:** *Eimeria acervulina*, RNA seq, dynamic development, transcriptome

## Abstract

Coccidiosis, a parasitic disease caused by single or multiple *Eimeria* species, leads to significant economic losses in the poultry industry. The *Eimeria* life cycle includes schizogony, gametogony, and sporogony. To investigate the dynamics of gene expression and regulatory networks during the development of *Eimeria acervulina*, we employed time-course transcriptomics to rigorously compare the gene expression patterns between a precocious line (PL) and the wild type (WT) of *E. acervulina*. The results revealed that the PL enters into gametogony 12 h earlier than the WT, and both the PL and WT exhibited distinct clustering patterns during the development phase. A weighted gene co-expression network analysis (WGCNA) identified genes specifically expressed at four distinct developmental stages, schizogony, gametogony, sporulated oocysts, and unsporulated oocysts, clarifying the key biological processes at each stage. This study used global transcriptome profiling to elucidate molecular variations throughout the *E. acervulina* life cycle, providing critical insights into molecular characterization and valuable resources for investigating other apicomplexan parasites of public health importance.

## 1. Introduction

*Eimeria* is a genus of intracellular protozoan parasites, with seven predominant species and three newly discovered species, known to cause coccidiosis in poultry [1]. *E. acervulina* is widely distributed in the poultry industry and is considered one of the most pathogenic species with a high prevalence in infected chickens [2]. Infection at high doses results in health issues such as reduced growth rate, weight loss, and a low feed conversion ratio in poultry, significantly diminishing the profitability of the poultry industry [3]. Chicken coccidiosis reportedly accounted for a global cost of ~£13.0 billion, including reduced productivity like reduced body weight and increased costs for prophylactic control, vaccination, and therapeutic treatment [4]. However, the emergence of drug-resistant strains and the presence of drug residues have increasingly limited the efficacy and selection of anticoccidial medications [5]. The identification of novel targets and antigenic molecules for coccidiosis treatment is an urgent priority, requiring a deeper comprehension of the biological mechanisms of coccidia.

The life cycle of *E. acervulina* encompasses three critical stages: schizogony, gametogony, and sporogony. In particular, precocious lines (PLs), characterized by a reduced number of schizogonic generations or accelerated developmental rate, are stably heritable and have thus become ideal materials for studying development-related genes [6]. Complex development stages of *E. acervulina* are consistent with its stage-specific characteristics of genetics, including specifically expressed genes and proteins, which could provide fundamental information in the discovery of effective vaccine and drugs against coccidiosis. Previous studies have predominantly relied on morphological techniques, such as electron microscopy, to describe the various developmental stages of *Eimeria* and the growth characteristics of PLs compared to the WT [7,8,9,10]. However, due to the asynchronous nature of coccidian development, these findings are significantly influenced by subjective judgment, particularly during the first generation of schizogony, where the scarcity and uneven distribution of *Eimeria* can lead to observations being biased by sampling location.

Extensive transcriptome analyses have shown that the apicomplexan parasites *Plasmodium* and *Toxoplasma gondii* primarily regulate the expression of functional gene products in a stage-specific manner through epigenetic and transcriptional mechanisms. Quantitative profiling of gene transcription has led to the identification of stage-specific genes in the sporozoite and oocyst stages of *T. gondii* [11,12], as well as the sexual differentiation of *P. falciparum* [13]. The gene-encoding Apical Membrane Antigen 1 (AMA1), which is specifically expressed in the oocysts of *T. gondii* and plays a conserved role in invasion across apicomplexan parasites, suggests that AMA1-based vaccines may offer multi-stage protection against various apicomplexan parasites [14]. Additionally, the ApiAP2 family has been recognized as the most populous family of transcription factor regulators of *P. falciparum* [15]. Specifically, AP2-L (PF3D7_0730300) has been confirmed as the specific regulator associated with liver-stage development. Functional studies have demonstrated that the ablation of AP2-L leads to a significant attenuation in hepatic infectivity, with an approximate 10,000-fold decrease in liver infection capacity [16], while in *Eimeria* spp., only a limited number of stage-specific genes have been identified in the oocysts and merozoites of *Eimeria tenella*, *Eimeria maxima*, and *Eimeria necatrix* [17,18,19]. Moreover, previous studies selected a limited number of sampling times, and there are no studies that have been reported that analyze the complete transcriptome of *E. acervulina* throughout its life cycle, nor its stage-specific genes. In our research, we conducted a time-course comparative transcriptomics study across 17 time points, revealing gene expression patterns throughout the developmental stages of *E. acervulina* and identifying key transcription factors that regulate transitions between these stages. This comparative approach has enhanced our understanding of the *E. acervulina* life cycle, potentially offering key molecular targets for advancing prevention and control strategies against coccidiosis.

## 2. Materials and Methods

### 2.1. Animals and Parasites

Four-day-old Arbor Acres (AA) chickens (*n* = 102) were purchased from Merial Animal Health Co., Ltd. (Beijing, China) and fed a coccidia-free diet and water. The PL and WT of *E. acervulina* were maintained at the National Animal Protozoa Laboratory, College of Veterinary Medicine, China Agricultural University, Beijing, China. We assert that all procedures complied with the ethical standards of the relevant national and institutional guides on the care and use of laboratory animals, and all the experiments were reviewed and approved by the China Agricultural University Animal Ethics Committee and Beijing Laboratory Animal Experimental Ethical Inspection(ID: AW52803202-2-1).

### 2.2. Preparation of Samples for RNA-Seq

The chickens were divided into seventeen groups, with each group consisting of six chickens. In each group, three chickens were inoculated with 5 × 10^6^ sporulated oocysts of WT *E. acervulina*, while the other three were inoculated with 5 × 10^6^ sporulated oocysts of PL *E. acervulina*. Duodenal tissue samples were collected at fifteen time points post-inoculation (6, 12, 18, 24, 36, 48, 60, 72, 84, 96, 108, 120, 132, 144, and 156 h). Unsporulated oocysts (USOs) were collected from the duodenal contents at 7 days post-infection (dpi) and subsequently purified. Sporulated oocysts (SOs) were collected from the feces at 8 dpi and then subjected to sporulation at 28 °C in a 2.5% potassium dichromate solution for 48 h. All the samples were frozen in liquid nitrogen and stored at −80 °C for subsequent RNA extraction and sequencing.

### 2.3. RNA Sequencing and Bioinformatic Analyses

Total RNA was extracted with Trizol Reagent (Invitrogen Life Technologies, Carlsbad, CA, USA), and the concentration, quality, and integrity of RNA were assessed with a NanoDrop spectrophotometer (Thermo Scientific, Waltham, MA, USA). Then, the purified high-quality RNA was used to construct a sequencing library using an NEBNext^®^ Ultra™ RNA Library Prep Kit for Illumina^®^ (NEB, Ispawich, CA, USA). The sequencing library was sequenced on the NovaSeq 6000 platform (Illumina) by Shanghai Personal Biotechnology Co., Ltd. (Shanghai, China). The raw data output of 150 bp paired-end reads were trimmed using Trimmomatic, version 0.36, with default parameters to filter connectors and low-quality reads for subsequent analysis [20]. Then, the high-quality clean reads were aligned to a chromosome-level genome of *E. acervulina*, previously assembled by our lab using STAR mapper, version 2.7.2b (unpublished data) [21]. Read counting was performed using featureCounts, and then transcript abundance in transcripts per million (TPM) was calculated from featureCounts output [22]. A sample correlation matrix was generated by calculating the Spearman correlation coefficient (SCC) for each possible pair of samples, using the R package ‘pheatmap’. Principal component analysis (PCA) was performed using the PCAtools to exhibit the correlation of transcriptomes (https://github.com/kevinblighe/PCAtools, accessed on 19 June 2024) [23]. Weighted Gene Go-expression Network Analysis (WGCNA) was performed using the R package “WGCNA” to identify modules of highly correlated genes, summarize the interconnections between modules and associations with external sample traits, and identify candidate biomarkers or therapeutic targets. The optimal soft threshold was chosen to convert the correlation matrix into an adjacency matrix, and a topological overlap matrix (TOM) was created from the adjacency matrix. The TOM-based phase dissimilarity metric was utilized to categorize genes with similar expression patterns into gene modules using average linkage hierarchical clustering. Subsequently, Gene Ontology (GO) enrichment analysis for genes of interest was performed using the *Phyper* function in R software (version 2.12.1). An adjusted *p*-value of ≤0.01 was considered to indicate significant enrichment. All annotation procedures were conducted for *E. acervulina*.

## 3. Results

### 3.1. Similar but Accelerated Gene Expression Dynamics in the Precocious Line of E. acervulina

**Figure 1:**
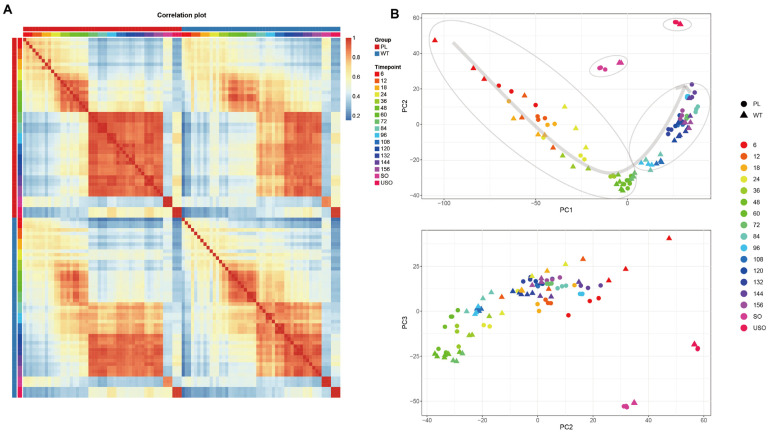
Transcriptomic profiles of precocious line (PL) and wild type (WT) of *E. acervulina* at different developmental time points. (**A**) Heatmap of transcriptomic correlations between the precocious line (PL) and wild type (WT) of *E. acervulina* at various developmental stages. The top row in red represents the PL group, and in blue, the WT group, with the second row indicating the different infection times from 6 to 156 h. (**B**) Principal Component Analysis (PCA) of different developmental stages of PL and WT. Each point represents a sample, with PC1 typically associated with the greatest variance among samples and PC2 revealing secondary patterns of variation. Colors and shapes in the figure are used to differentiate between the various sample groups, with circles representing PL and triangles representing WT.

To assess the internal consistency of RNA sequencing data, we conducted Pearson correlation analysis and principal component analysis (PCA) on the samples, and the results revealed a high degree of concordance among biological replicates. Both the PL and WT exhibited distinct clustering patterns during the endogenous development phase. Clustering in the PL occurred between 60 and 72 h post-inoculation (h.p.i.), whereas WT was observed between 72 and 84 h.p.i. This is consistent with the histological section analysis, which demonstrated that this period corresponds to the transition from asexual to sexual reproduction (Figure 1A, Supplementary Figure S1). The transcriptomes of PLs appear to be expressed earlier than those in WTs; Spearman’s correlation of transcriptomes between PLs and WTs is nearly 0.79, indicating that the accelerated expressions in PLs could be strongly related to the precocious trait. The PCA results further validated that the principal disparities between PL and WT were attributable to developmental stage rather than strain variation and revealed a clear temporal correlation in gene expression, delineating distinct clusters associated with the developmental stages of schizogony, gametogony, unsporulated oocysts, and sporulated oocysts (Figure 1B).

**Figure 2:**
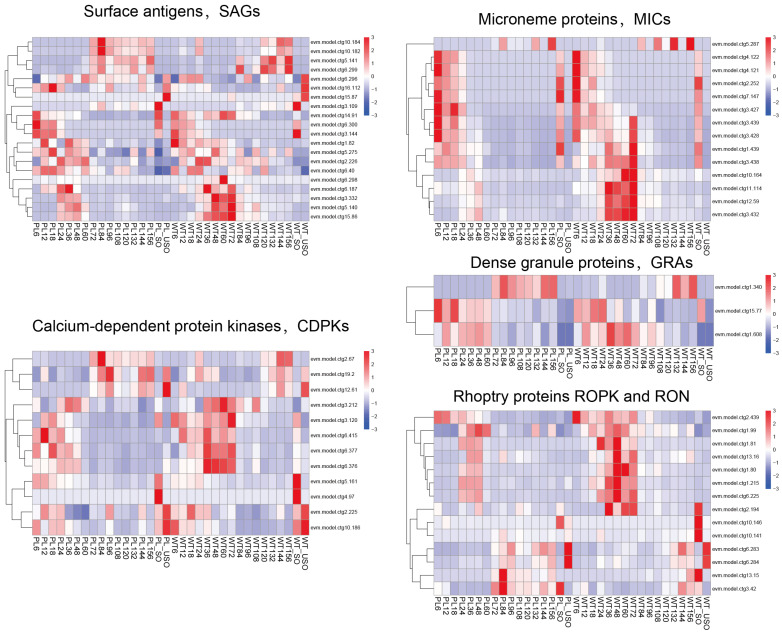
Heatmap of key gene expression during host cell invasion of the precocious line (PL) and wild-type (WT) *E. acervulina* at various time points. The heatmap illustrates the expression distribution of genes encoding for Rhoptry proteins (ROPs), Rhoptry neck proteins (RONs), Surface antigens (SAGs), Microneme proteins (MICs), Dense granule proteins (GRAs), and Calcium-dependent protein kinases (CDPKs) throughout the endogenous development timeline, as well as in sporulated and un-sporulated oocysts. Gene expression levels were normalized after calculating the Z-Score.

Additionally, a comparative analysis of the expression levels of key molecules involved in the invasion of host cells by *Eimeria*, including rhoptry proteins, neck proteins, surface antigens, microneme proteins, dense granule antigens, and calcium-dependent protein kinase genes, showed that these invasion-associated genes exhibited stage-specific expression patterns throughout the developmental process of the parasite. Significant differences in their expression levels were observed, particularly during the transition from asexual to sexual reproduction, as well as during the stages of unsporulated and sporulated oocysts (Figure 2). These functionally important genes also showed advanced but similar expression patterns in the PL compared to the WT.

### 3.2. Key Regulatory Pathways in E. acervulina Developmental Stages Identified through Comparative Gene Expression Analysis

**Figure 3:**
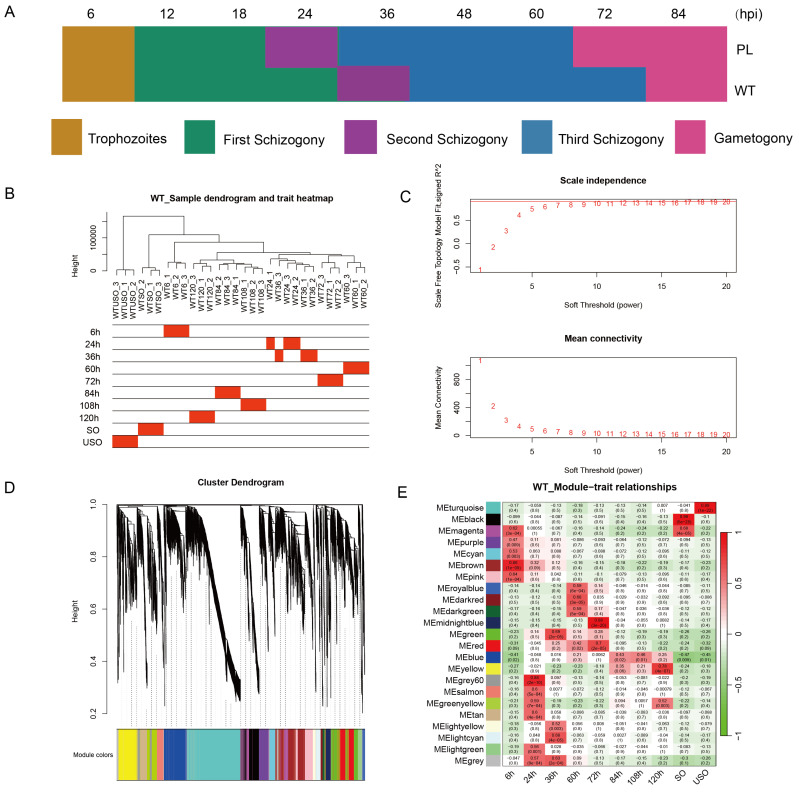
Identification of gene modules associated with representative developmental time points in the WT of *Eimeria acervulina*. (**A**) An endogenous developmental schematic of the precocious strain (PL) and wild type (WT) of *E. acervulina* based on transcriptomic results. (**B**) Clustering characteristics of the wild type (WT) of *E. acervulina* at various developmental time points. (**C**) Determination of the optimal β value through a comparison of scale-free topology and average connectivity, with a filtering criterion of R-square = 0.85. Inspection of scale-free topology at a soft threshold of 10. (**D**) Hierarchical clustering dendrogram of all expressed genes with assigned module colors. (**E**) Heatmap of the correlation and significance between developmental time points and gene modules. The color bar on the left indicates the different classifications of the identified gene modules.

**Figure 4:**
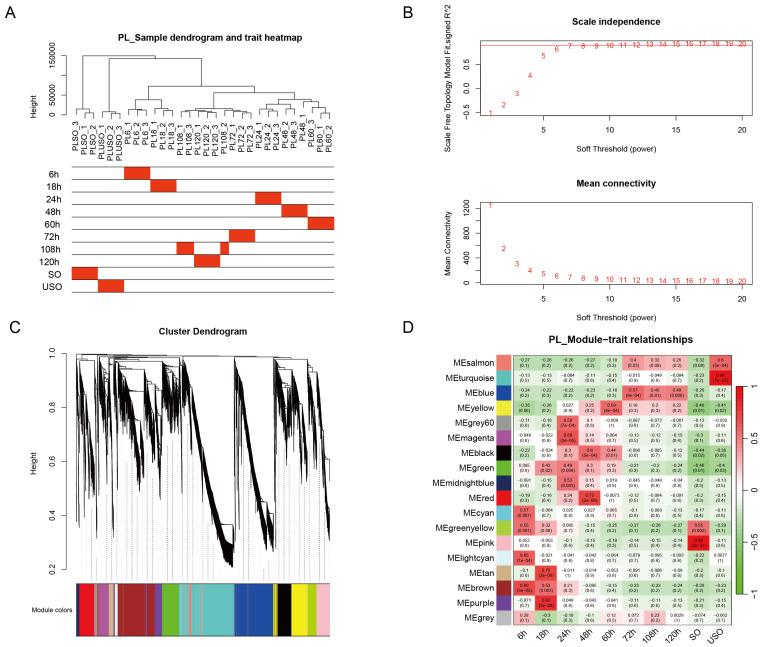
Identification of gene modules associated with representative developmental time points in PL of *E. acervulina*. (**A**) Clustering characteristics of the precocious line (PL) of *E. acervulina* at various developmental time points. (**B**) Determination of the optimal β value through a comparison of scale-free topology and average connectivity, with a filtering criterion of R-square = 0.85. Inspection of scale-free topology at a soft threshold of 7. (**C**) Hierarchical clustering dendrogram of all expressed genes with assigned module colors. (**D**) Heatmap of the correlation and significance between developmental time points and gene modules. The color bar on the left indicates the different classifications of the identified gene modules.

To further elucidate the key genes influencing the developmental process, we selected ten critical time points during the development of both PL and WT for Weighted Gene Co-expression Network Analysis (WGCNA). For the WT, the chosen time points encompassed developmental stages at 6, 24, 36, 60, 72, 84, 108, and 120 h.p.i, as well as the unsporulated oocyst (USO) and sporulated oocyst (SO) stages (Figure 3). Owing to the accelerated development characteristic of PL, the analysis commenced at an earlier time point of 18 h. Similarly, ten key time points throughout the developmental process were selected (6, 18, 24, 48, 60, 72, 108, 120 h.p.i, as well as the USO and SO). Correlation analysis was performed between the time point and a scale-free expression network, revealing the gene modules most strongly associated with each time point. Based on the correlation coefficients and *p*-values of the modules, we identified the highly correlated gene modules that represent the developmental characteristics of each time point (Figure 4).

**Figure 5:**
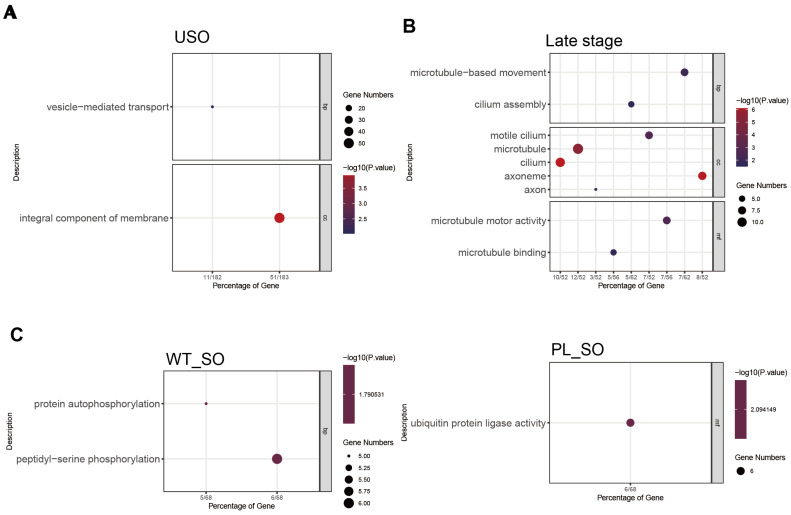
Gene functional clustering analysis during the sporulation, un-sporulation, and late sexual reproduction stages of *E. acervulina*. (**A**) Results of the significant Gene Ontology (GO) functional enrichment analysis for representative genes shared between the precocious and wild-type (WT) *E. acervulina* during the un-sporulation stage. (**B**) Results of the significant GO functional enrichment analysis for genes shared between the precocious and wild-type (WT) *E. acervulina* during the late sexual reproduction stage. (**C**) Significant GO functional enrichment analysis for module genes corresponding to the sporulation stage in the wild-type (WT) and precocious *E. acervulina* strains, respectively.

**Figure 6:**
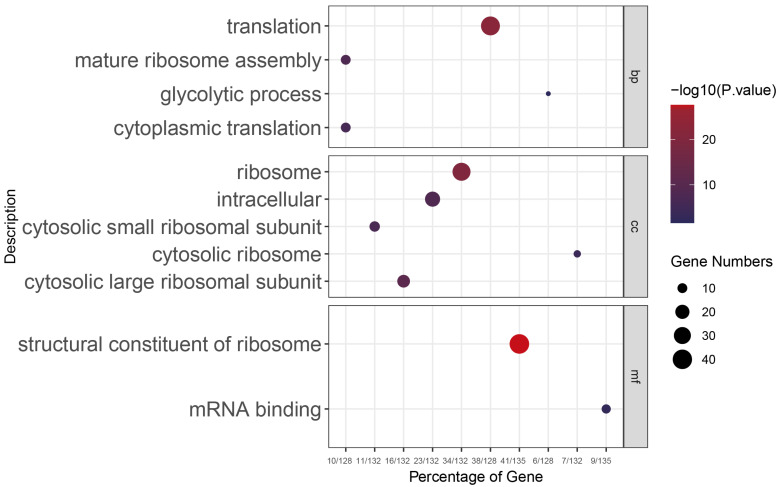
Gene functional clustering analysis at schizogony of *E. acervulina*.

A comparative analysis of unsporulated oocyst (USO)-associated genes between the PL and WT identified 612 commonly expressed genes. A GO enrichment analysis of these shared genes highlighted significant enrichment in the ‘Vesicle-mediated transport’ pathway, predominantly associated with the ‘Integral component of membrane’ (Figure 5A). Examination of the sporulated oocyst (SO) stage revealed that the PL showed enrichment in the ‘Ubiquitin protein ligase activity’ pathway, in contrast to the WT, which exhibited enrichment in the ‘Protein autophosphorylation’ and ‘Peptidyl-serine phosphorylation’ pathways. These pathways, ubiquitination, and autophosphorylation are integral post-translational modification (PTM) processes that regulate protein function, stability, and cellular signaling (Figure 5C). An analysis of gene expression patterns at later stages identified 262 enriched genes following sexual reproduction, correlating with microtubule formation and ciliary movement functions (Figure 5B). This included known female gametocyte-specific (GAM56) and male gametocyte-specific (HAP2) genes. WGCNA results allowed us to pinpoint gene clusters with the highest correlation to each developmental time point. Given the considerable number of functionally uncharacterized genes in coccidia, not every schizogony stage time point demonstrated significant functional enrichment. Functional enrichment at the 60 h time point, corresponding to the schizogony stage, indicated that the parasite was significantly engaged in ribosome biogenesis, cell proliferation, and differentiation processes (Figure 6).

**Figure 7:**
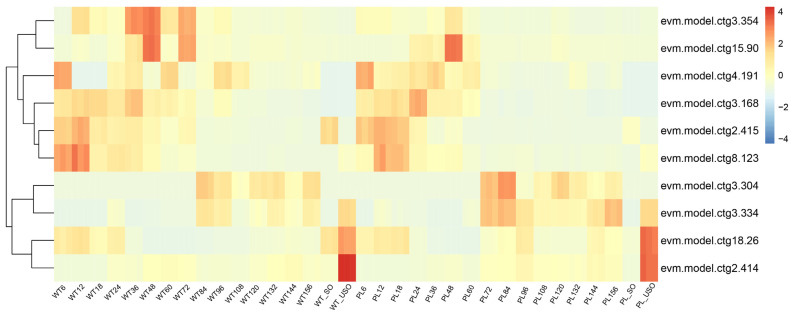
Heatmap of AP2 gene expression of the precocious line (PL) and wild-type (WT) *E. acervulina* at various time points.

Comparative analysis of the USO modules between the PL and WT revealed that two genes containing AP2 domains, designated as evm.model.ctg18.26 and evm.model.ctg2.414, exhibited increased expression levels in the USO. Additionally, genes evm.model.ctg3.304 and evm.model.ctg3.334 showed elevated expression during the sexual development transition phase in both parasite strains, with expression occurring 12 h earlier in the PL compared to the WT (Figure 7). We further investigated the interactions between gene sets at the sexual reproduction and USO stages with AP2 genes. The results showed that during the sexual reproduction phase, genes with significant correlation coefficients (all exceeding 0.7) with evm.model.ctg3.304 and evm.model.ctg3.334 encoded functions such as microtubule binding and cilium in PL, and these genes were also associated with the formation of sperm and intraflagellar transport in WT (Supplementary Tables S1 and S2). The two genes, evm.model.ctg2.414 and evm.model.ctg18.26, which belong to the AP2 family, were identified during the USO stage. Genes exhibiting a high correlation with these two genes were involved in the regulation of proteins and lipids, as well as in the repair of membrane structures, all of which were related to the mechanisms of vesicle-mediated transport. (Supplementary Tables S3 and S4).

## 4. Discussion

We presented, for the first time, high-quality gene expression profiles in the development of the PL and WT of *E. acervulina* using dual RNA-Seq analysis. Following a comprehensive transcriptomic analysis, we identified key regulatory pathways and transcription factors that may be associated with sexual development and the unsporulated oocyst stage. Through comparative analysis of the transcriptomic profiles between the PL and WT, we observed similar expression patterns between the two strains. The expression dynamics of invasion-associated genes indicated that their transcription in the PL occurred 12 h earlier than in the WT. Prior research has indicated that different species of *Eimeria* have variations in the quantity of invasion-associated proteins [24,25,26,27]. These proteins exhibit specific expression during the sporozoite, unsporulated oocyst, and schizogony stages, providing a rich resource for the discovery of candidate antigenic molecules [28,29].

In a previous investigation of *Eimeria*, Su et al. used the developmental transition characteristic of *E. necatrix*, where the first and second generations of schizogony occur within the mid-jejunum, shifting to cecal epithelial cells at the third generation of schizogony. Using transcriptome sequencing, Su et al. suggested that second-generation schizont genes are significantly engaged in processes such as ribosome synthesis, DNA replication, and RNA transport—aligning with functional enrichments observed in our study [30]. These processes are intricately linked to growth and reproductive capacities, indicating the importance of ribosomal biogenesis in cellular proliferation and differentiation. Contrasting with the gametocyte stage, Su et al. revealed that genes active during the transition to sexual reproduction are predominantly involved in ribosome biogenesis, the HIF-1 signaling pathway, and the pentose phosphate pathway, all of which are implicated in material and energy metabolism. It is postulated that this energy storage may be a strategic preparation for the subsequent sporogony phase in *E. necatrix* [31]. Walker et al. revealed that the gametocytes of *E. tenella* code for the proteins involved in oocyst wall biosynthesis and fertilization [32]. Despite identifying stage-specific genes, functional research on *Eimeria* species remains incomplete. Future research must employ advanced techniques to understand their developmental roles, which will aid in identifying molecular targets for control strategies.

## 5. Conclusions

We comprehensively conducted transcriptomic analysis of gene expression and associated biological processes of the PL and WT of *E. acervulina* at 17 distinct time points. By comparing the gene expression patterns between the two strains during their shared developmental phases, we identified specific genes and their functions at various developmental stages. The findings provide novel insights into the gene regulatory networks during the development of *E. acervulina*. The results not only enrich our understanding of coccidian biology but also offer potential molecular targets and a theoretical foundation for future vaccine development and drug screening.

## Data Availability

The RNA-Seq data for these samples are available in the National Genomics Data Center, under accession number subPRO038927. This study did not generate any custom code. Any additional information required to reanalyze the data reported in this article is available from the lead contact on request.

## Supplementary Materials

The following supporting information can be downloaded at: https://www.mdpi.com/article/10.3390/genes15070831/s1, Figure S1: Microscopic observations of endogenous development of WT (top lines) and PL (bottom lines) in duodenum. Bar = 10 μm; Table S1: Correlation Analysis and Functional Description of AP2 Transcription Factors with Other Genes in the Sexual development Transition Module of PL; Table S2: Correlation Analysis and Functional Description of AP2 Transcription Factors with Other Genes in the Sexual development Transition Module of WT; Table S3: Correlation Analysis and Functional Description of AP2 Transcription Factors with Other Genes in the unsporulated oocyst Module of PL; Table S4: Correlation Analysis and Functional Description of AP2 Transcription Factors with Other Genes in the unsporulated oocyst Module of WT.

## References

[1] Blake D.P., Vrba V., Xia D., Jatau I.D., Jatau I.D., Spiro S., Nolan M.J., Underwood G., Tomley F.M. (2021). Genetic and biological characterization of three cryptic *Eimeria* operational taxonomic units that infect chickens (*Gallus gallus* domesticus). Int. J. Parasitol..

[2] Gao Y., Sun P., Hu D., Tang X., Zhang S., Shi F., Yan X., Yan W., Shi T., Wang S. Advancements in understanding chicken coccidiosis: From *Eimeria* biology to innovative control strategies. One Health Adv..

[3] Blake D.P., Worthing K., Jenkins M.C. (2020). Exploring *Eimeria* Genomes to Understand Population Biology: Recent Progress and Future Opportunities. Genes.

[4] Blake D., Knox J., Dehaeck B., Huntington B., Rathinam T., Ravipati V., Ayoade S., Gilbert W., Adebambo A.O., Jatau I.D. (2020). Re-Calculating the cost of coccidiosis in chickens. Vet. Res..

[5] Saeed Z., Alkheraije K.A. (2023). Botanicals: A promising approach for controlling cecal coccidiosis in poultry. Front. Vet. Sci..

[6] Gu X., Liu H., Li C., Fang S., Cui P., Liao Q., Zhang S., Wang S., Duan C., Yu F. (2019). Selection and characterization of a precocious line of *Eimeria media*. Parasitol. Res..

[7] Ball S.J., Pittilo R.M., Joyner L.P., Norton C.C. (1981). Scanning and transmission electron microscopy of *Eimeria maxima* microgametogenesis. Parasitology.

[8] Novilla M.N., Jeffers T.K., Griffing W.J., White S.L. (1987). A redescription of the life cycle of *Eimeria mitis* Tyzzer, 1929. J. Protozool..

[9] Kawazoe U., Bordin E.L., de Lima C.A., Dias L.A. (2005). Characterisation and histopathological observations of a selected Brazilian precocious line of *Eimeria acervulina*. Vet. Parasitol..

[10] Vetterling J.M., Doran D.J. (1966). Schizogony and gametogony in the life cycle of the poultry coccidium, *Eimeria acervulina* Tyzzer, 1929. J. Parasitol..

[11] Behnke M.S., Zhang T.P., Dubey J.P., Sibley L.D. (2014). *Toxoplasma gondii* merozoite gene expression analysis with comparison to the life cycle discloses a unique expression state during enteric development. BMC Genom..

[12] Fritz H.M., Buchholz K.R., Chen X., Durbin-Johnson B., Rocke D.M., Conrad P.A., Boothroyd J.C. (2012). Transcriptomic analysis of *Toxoplasma* development reveals many novel functions and structures specific to sporozoites and oocysts. PLoS ONE.

[13] Silvestrini F., Bozdech Z., Lanfrancotti A., Giulio E.D., Bultrini E., Picci L., deRisi J.L., Pizzi E., Alano P. (2005). Genome-wide identification of genes upregulated at the onset of gametocyte genesis in *Plasmodium falciparum*. Mol. Biochem. Parasitol..

[14] Santos J.M., Ferguson D.J., Blackman M.J., Soldati-Favre D. (2011). Intramembrane cleavage of AMA1 triggers *Toxoplasma* to switch from an invasive to a replicative mode. Science.

[15] Modrzynska K., Pfander C., Chappell L., Yu L., Suarez C., Dundas K., Gomes A.R., Goulding D., Rayner J.C., Choudhary J. (2017). A Knockout Screen of ApiAP2 Genes Reveals Networks of Interacting Transcriptional Regulators Controlling the *Plasmodium* Life Cycle. Cell Host Microbe.

[16] Iwanaga S., Kaneko I., Kato T., Yuda M. (2012). Identification of an AP2-family protein that is critical for malaria liver stage development. PLoS ONE.

[17] Amiruddin N., Lee X.W., Blake D.P., Suzuki Y., Tay Y.L., Lim L.S., Tomley F.M., Watanabe J., Sugimoto C., Wan K.-L. (2012). Characterisation of full-length cDNA sequences provides insights into the *Eimeria tenella* transcriptome. BMC Genom..

[18] Matsubayashi M., Hatta T., Miyoshi T., Anisuzzaman S.K., Shimura K., Isobe T., Kita K., Tsuji N. (2013). High-throughput RNA sequencing profiles and transcriptional evidence of aerobic respiratory enzymes in sporulating oocysts and sporozoites of *Eimeria tenella*. Infect. Genet. Evol..

[19] Novaes J., Rangel L.T., Ferro M., Abe R.Y., Manha A.P., de Mello J.C., Varuzza L., Durham A.M., Madeira A.M.B., Gruber A. (2012). A comparative transcriptome analysis reveals expression profiles conserved across three *Eimeria* spp. of domestic fowl and associated with multiple developmental stages. Int. J. Parasitol..

[20] Bolger A.M., Lohse M., Usadel B. (2014). Trimmomatic: A flexible trimmer for Illumina sequence data. Bioinformatics.

[21] Dobin A., Davis C.A., Schlesinger F., Drenkow J., Zaleski C., Jha S., Batut P., Chaisson M., Gingeras T.R. (2013). STAR: Ultrafast universal RNA-seq aligner. Bioinformatics.

[22] Liao Y., Smyth G.K., Shi W. (2014). featureCounts: An efficient general purpose program for assigning sequence reads to genomic features. Bioinformatics.

[23] Blighe K., Lun A. (2019). PCAtools: Everything Principal Components Analysis. https://github.com/kevinblighe/PCAtools.

[24] Li C., Zhao Q., Zhu S., Wang Q., Wang H., Yu S., Yu Y., Liang S., Zhao H., Huang B. (2020). *Eimeria tenella* Eimeria-specific protein that interacts with apical membrane antigen 1 (EtAMA1) is involved in host cell invasion. Parasites Vectors.

[25] Zhao N., Ming S., Sun L., Wang B., Li H., Zhang X., Zhao X. (2021). Identification and Characterization of *Eimeria tenella* Microneme Protein (EtMIC8). Microbiol. Spectr..

[26] Augustine P.C. (2001). Cell: Sporozoite interactions and invasion by apicomplexan parasites of the genus *Eimeria*. Int. J. Parasitol..

[27] Gao Y., Suding Z., Wang L., Liu D., Su S., Xu J., Hu J., Tao J. (2021). Full-length transcriptome analysis and identification of transcript structures in *Eimeria necatrix* from different developmental stages by single-molecule real-time sequencing. Parasites Vectors.

[28] Kim S.K., Boothroyd J.C. (2005). Stage-specific expression of surface antigens by *Toxoplasma gondii* as a mechanism to facilitate parasite persistence. J. Immunol..

[29] Zhang Z., Li Y., Liang Y., Wang S., Xie Q., Nan X., Li P., Hong G., Liu Q., Li X. (2018). Molecular characterization and protective immunity of rhoptry protein 35 (ROP35) of *Toxoplasma gondii* as a DNA vaccine. Vet. Parasitol..

[30] Su S., Hou Z., Liu D., Jia C., Wang L., Xu J., Tao J. (2017). Comparative transcriptome analysis of second- and third-generation merozoites of *Eimeria necatrix*. Parasites Vectors.

[31] Su S., Hou Z., Liu D., Jia C., Wang L., Xu J., Tao J. (2018). Comparative transcriptome analysis of *Eimeria necatrix* third-generation merozoites and gametocytes reveals genes involved in sexual differentiation and gametocyte development. Vet. Parasitol..

[32] Walker R.A., Sharman P.A., Miller C.M., Lippuner C., Okoniewski M., Eichenberger R.M., Ramakrishnan C., Brossier F., Deplazes P., Hehl A.B. (2015). RNA Seq analysis of the *Eimeria tenella* gametocyte transcriptome reveals clues about the molecular basis for sexual reproduction and oocyst biogenesis. BMC Genom..

