# Supplementary figures and images for: Transcriptomic Insights into the Developmental Dynamics of Eimeria acervulina: A Comparative Study of a Precocious Line and the Wild Type
Source: Genes (Basel). 2024 Jun 24;15(7):831. doi: 10.3390/genes15070831 (PMC11276583; doi:10.3390/genes15070831)

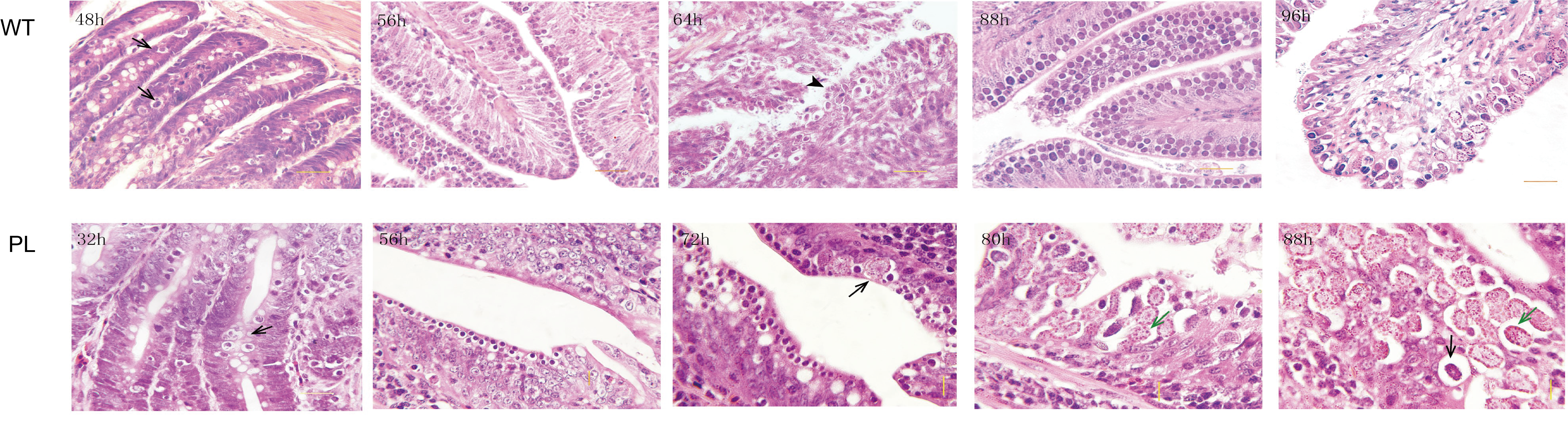

Supplement: Supplementary file 1 [file genes-15-00831-s001.zip › Supplementary FigS1.png]
